# The impact of an enteral formula with food‐derived ingredients on dietetic practice at a specialist children's hospital in the UK: Retrospective study

**DOI:** 10.1111/jhn.13374

**Published:** 2024-10-16

**Authors:** Graeme O'Connor, Angela Camacho Velandia, Zoltan Hartfiel Capriles

**Affiliations:** ^1^ Department of Dietetics Great Ormond Street Hospital Foundation Trust London UK; ^2^ Department of Biometry Statistics Sofpromed Clinical Investigation Palma de Mallorca Spain

**Keywords:** acute setting, blended diet, children, enteral nutrition, food‐based enteral formula, food derived ingredients, gastrointestinal symptoms

## Abstract

**Background:**

Blended tube feeds are reported to be better tolerated in some children compared to standard commercial enteral formulas, allowing children to normalise feeding by having similar foods as the rest of the family. However, a blended tube feed is contraindicated in patients who are immunocompromised or require post‐pyloric feeding as a result of a food safety risk. Other contraindications for blended diet include children who require continuous pump feeding via gastrostomy or nasogastric feeding tube (< 12 Fr) and fluid restrictions. To meet the demands of consumers, manufacturers have developed enteral formulas with food‐derived ingredients (EFI). Commercially available EFIs are relatively novel in the UK. The present study aimed to monitor the implementation of an EFI by dietitians in a specialist children's hospital.

**Methods:**

A single‐centre retrospective study was conducted to monitor the dietetic practice of commencing a commercially available EFI (Compleat® paediatric; Nestlé Health Science; 1.2 kcal/ml with 14% food‐derived ingredients). Using electronic medical notes, data were collected on all children who commenced an EFI via an enteral feeding tube in a specialist paediatric hospital between August 2022 and December 2023. Data were gathered on demographics (age, sex and primary diagnosis), anthropometric measurements (weight‐for‐age *Z*‐score and height‐for‐age *Z*‐score), feed regimens (feed volume, feeding route, mode of feeding [continuous, bolus]), gastrointestinal symptoms (gastro‐oesophageal reflux, vomiting, abdominal discomfort, constipation and loose stools) and geographical discharge area for children on home enteral nutrition.

**Results:**

Seventy children were included in the analysis. The mean ± SD age was 4.7 ± 6 years. The median admission weight‐for‐age *Z*‐score was −1.50. The most common primary diagnosis was a neurological impairment in 37/70 (47%) children. Most children were fed via a percutaneous endoscopic gastrostomy 31/70 (44%) and 8/70 (11%) of the children fed directly into the jejunum. The most common reason being gastrointestinal symptoms, 58/70 (83%). The most common gastrointestinal symptom reported before commencing an EFI was loose stools in 22/58 (38%) children. Within 7 days of commencing an EFI, there was reported improvement in gastrointestinal symptoms in all categories. In total, 42/70 children were discharged on an EFI.

**Conclusions:**

In our specialist children's hospital, EFI is primarily implemented by dietitians in children who are already established on an enteral formula displaying gastrointestinal symptoms. However, dietitians are increasingly implementing an EFI as their first‐line whole protein enteral formula. Furthermore, an EFI was also implemented as a compromise to a blended diet.

## Key points

- Enteral formulas with food‐derived ingredients are a new addition to the commercially available enteral formulas in the UK.
- Enteral formulas with food‐derived ingredients are being implemented in children established on tube feeding who displaying gastrointestinal symptoms.
- Enteral formulas with food‐derived ingredients are well tolerated and improve gastrointestinal symptoms

## INTRODUCTION

Commercially available enteral nutrition has come full circle in the last 50 years. Before 1973, when the first nutrient‐intact formula came on the market, enteral nutrition consisted of blended foods.^1^ The use of commercial enteral formulas became standard practice in hospitals because of the decreased risk of microbial contamination and ease of administration.^1^ There has been a consumer shift with trends in food selection and consumption to whole food, organically grown and locally sourced with perceived health benefits. This health‐conscious trend has filtered through to paediatric home enteral nutrition, with an increased desire of parents/carers to prepare homemade food blends.^2^ There are many reasons for the use of homemade blended enteral feeds, including better tolerance than commercially available enteral formulas, perception of nutrition benefits from homemade food as a result of the variety of food items and the option of tailoring to special needs.^3^ Psychosocial aspects also have an influence; for example, the desire to feed one's child and serve the same foods as the rest of the family.^4^

Homemade blended feeds allow clinicians and patients/caregivers the opportunity to customise the formula to meet the macronutrient and micronutrient requirements of the consumer at the same time as synchronising the patient's preferences for ‘food choices’ based on cultural, religious, ethical and individual health concerns.^5^ Initiation and maintenance of blended tube feeds are associated with improved clinical outcomes including reductions in gastrointestinal symptoms, hospitalisations and increased intestinal bacterial diversity.^6^

The American Society for Parental and Enteral Nutrition (ASPEN),^7^ European Society of Parenteral Hepatology Gastroenterology and Nutrition (ESPHGAN)^8^ and the British Dietetic Association have developed practice guidance on the use of blended tube feed. A review by Peers et al.^9^ examined the impact of an enteral blended feed compared to commercial feeds, on gastrointestinal symptoms of adults and children who are tube‐fed. The review reported that diarrhoea is prevalent in tube‐fed populations and associated with adverse outcomes. Improvements in diarrhoea symptoms attributed to blended tube feeds may be clinically important. Another review article by McCormack et al.^10^ synthesised the available evidence on the benefits and complications of blended tube feeds versus commercial feeds, stating there is a paucity of data in this area and much heterogeneity in the included studies, but the available literature points towards positive outcomes. The authors conclude that a blended diet for enteral nutrition is an important and highly relevant topic, and more primary research is required.

To meet the demands of consumers for blended tube feeds, manufacturers began marketing commercially prepared food‐based enteral formulas.^11^ In an acute clinical and community setting, a blended tube feed is contraindicated in patients who are immunocompromised or require post‐pyloric feeding as a result of a food safety risk.^7^, ^8^ Other contraindications for blended diet include children who require continuous pump feeding via gastrostomy or nasogastric feeding tube (< 12 Fr) and fluid restrictions.^12^, ^13^ Clinicians are responsible for prescribing the enteral tube feed best suited to meet the nutrient needs and metabolic demands of the patients; the “right formula for the right patient”.^1^

Commercially available EFIs are relatively novel in the UK and are currently limited to only one commercially available EFI, which was introduced to Great Ormond Street Children's Hospital in March 2021. Subsequently, there has been growing supportive evidence on the use of EFI in the UK. ^12^ This single‐centre retrospective study aimed to monitor the dietetic practice of implementing enteral formula with food‐derived ingredients in a specialist children's hospital.

## METHODS

### Study design and patient population

This was a single‐centre retrospective study to monitor the dietetic practice of commencing a commercially available EFI (Compleat® paediatric; Nestlé Health Science, Blancs, Switzerland; 1.2 kcal/ml with 14% food‐derived ingredients containing rehydrated chicken, peas and green beans, with peach puree and orange juice). We collected data for all children who commenced an EFI via an enteral feeding tube in a specialist paediatric hospital between August 2022 and December 2023. This is a follow‐up study from our published national multi‐centre study that collected data on gastrointestinal tolerance of an EFI from March 2021 to July 2022.^12^

Children's clinical and dietetic information was collected from the hospital's electronic records (EPIC; Epic Systems, Madison, WI, USA; and Electronic Dietetics Manager [EDM3000]; http://www.edm3000.com). We collected data on demographics (age, sex and primary diagnosis), anthropometric measurements (weight‐for‐age *Z*‐score and height‐for‐age *Z*‐score), feed regimens (feed volume, feeding route and mode of feeding [continuous, bolus]) and gastrointestinal symptoms (gastro‐oesophageal reflux, vomiting, abdominal discomfort [bloating/flatulence], constipation and loose stools). Data were not collected on medication or clinical status. All children who had started EFI were included in this review. When available, data were also collected on children who had started and had been receiving EFI for at least 1 month. Exclusion criteria included patients who did not commence an EFI. Data were inputted into a Microsoft Form (Microsoft, Redmond, WA, USA), which was automatically sent to Ixia Clinical Limited (Hertford, UK). Data were compiled and downloaded into an Excel sheet (Microsoft) for analysis performed by ZHC. The study was approved by the Great Ormond Street Hospital Audit, Quality Improvement and Service Evaluation Committee (registration number GOSH2022/3234).

### Clinical and nutrition procedures

All children received nutritional assessments from a paediatric dietitian during inpatient admission. Gastrointestinal symptoms had been documented in the electronic medical notes by the dietitian or medical team before and after the child commenced EFI. We categorised the reported change in gastrointestinal symptoms as either improved, no change or worsened after EFI was commenced on key markers of tolerance: (gastro‐oesophageal reflux, vomiting, abdominal discomfort [bloating/flatulence], constipation and loose stools). Constipation was defined by Rome IV Criteria as less than three defecations a week, as well as painful and hard stools.^14^ Loose stool was defined as more than one loose stool a day lasting longer than 7 days.^15^ Stool form scales are a standardised and inexpensive method of classifying stools into a finite number of categories that can be used by families and healthcare professionals. The Bristol Stool Scale is a visual stool form scale; the ideal stool is generally type 3 or 4 and easy to pass without being too watery. Types 1 and 2 indicate constipation, whereas types 6 and 7 indicate loose stools.^16^ Reflux was defined as the parental observation of the passage of gastric contents into the oesophagus causing regurgitation, posseting or vomiting, which leads to troublesome symptoms that affect daily functioning.^17^

### Anthropometric measurements

Weight and height were extracted from EPIC electronic growth charts. Of note, children with underlying limb or spine flexion deformities may have an inaccurate measurement. Assessment of height reflects adequate growth and nutritional status but can be challenging in children with malformations. The nutrition status weight‐for‐age and height‐for‐age was assessed using *Z*‐scores. ^18^ Moderate overweight and obesity were identified if the weight‐for‐age *Z*‐score were between +2 and +3 or above +3 SD, respectively. Conversely, underweight was identified as moderate and severe underweight if the Z‐scores were between −2 and −3 or below −3 SD, respectively.^19^

### Outcome measures

The primary outcome measure was the reason for the dietitian commencing an EFI. The secondary outcome measured tolerance to EFI within 7 days in relation to gastrointestinal symptoms, which were categorised into key markers of tolerance (gastro‐oesophageal reflux, vomiting, abdominal discomfort [bloating/flatulence], constipation and loose stools). Additional outcomes monitored how many children were discharged on an EFI, and the length of time (months) the child had been on an EFI at the time of data collection. If children were no longer under medical or dietetic care of the discharging hospital, we contacted the local hospital dietetic team to ascertain if the child was still having an EFI.

### Statistical analysis

Continuous data were tabulated using descriptive statistics (mean ± SD). To assess tolerance, medical and dietetic reports of upper and lower gastrointestinal symptoms before starting EFI (vomiting, abdominal pain, constipation and loose stools) were compared to reports within 7 days of starting EFI. Comparative analysis was used to compare change in weight‐for‐age *Z*‐score and nutritional intake after 1 month on an EFI. *p* < 0.05 was considered statistically significant. Statistical analysis was performed with SPSS, version 26 (IBM Corp., Armonk, NY, USA).

## RESULTS

### Demographics and feeding

In total, 70 children had commenced an EFI within the study period and were included in the analysis. Females accounted for 32/70 (46%) of children. The mean ± SD age was 4.7 ± 6 years, age category 4–6 years (33%) accounted for the highest group to be prescribed an EFI. The median admission weight‐for‐age *Z*‐score was −1.50 (4 SD). The most common primary diagnosis was a neurological disorder in 37/70 (53%) children. Most children were fed via a percutaneous endoscopic gastrostomy 31/70 (44%) and 8/70 (11%) children fed directly into the jejunum. Most children were fed continuously 41/70 (59%) compared to bolus feeding (38%) or a combination of continuous and bolus (3%) (Table 1).

**Table 1:** Demographic, primary diagnosis and feeding characteristics of children who commenced an enteral formula with food‐derived ingredients.

Sex, n (%)	
Female	32 (46)
Male	38 (54)
**Age category n, (%)**	
1–3 years	21 (30)
4–6 years	23 (33)
7–9 years	8 (11)
10–12 years	6 (9)
13–15 years	8 (11)
16–18 years	4 (6)
Mean ± SD	4.7 ± 6
**Weight‐for‐age Z‐score, mean ± SD**	−1.5 ± 4
**Height‐for‐age Z‐score, mean ± S**	−1.2 ± 2
**Primary diagnosis category, n (%)**	
Neurological	37 (53)
Cardiology	12 (17)
Gastrointestinal	5 (7)
Oncology	5 (7)
Metabolic	3 (4)
Renal disorder	3 (4)
Endocrine	2 (3)
ENT	2 (3)
Respiratory	1 (2)
**Route of feeding, n (%)**	
Percutaneous endoscopic gastrotomy (PEG)	36 (51)
Nasogastric	26 (37)
Nasojejunal	3 (4)
PEG‐Jejunal extension	3 (4)
Jejunostomy	2 (3)
**Delivery mode**	
Continuous	41 (59)
Bolus	27 (38)
Combined bolus and continuous	2 (3)

Abbreviation: ENT, ear, nose and throat.

Over 50% (37/70) of children in the present study had a neurological impairment, accounting for most children on long‐term gastrostomy feeding 65% (24/37). Children with neurological impairment also accounted for 50% (4/8) of children with post‐pyloric jejunal feeding (Table 2). Continuous feeding was the most implemented feeding mode in relation to the diagnosis category, except for cardiac, metabolic and oncology diagnosis categories, when bolus feeding was predominant (Table 3).

**Table 2:** Breakdown of the mode of feeding in relation to diagnosis category in children who commenced an enteral formula with food‐derived ingredients.

Diagnosis category, n (%)	PEG	Nasogastric	Naso‐jejunal	PEG Jejunal	Jejunostomy	Totals
Neurological	24 (65)	9 (24)		2 (5)	2 (5%)	37
Cardiac	3 (25)	8 (67)	1 (8)			12
Gastrointestinal	4 (80)		1 (20)			5
Oncology		5 (100)				5
Metabolic	1 (33)	1 (33)		1 (33)		3
Renal disorder		3 (100)				3
Endocrine	1 (50)		1 (50)			2
ENT	2 (100)					2
Respiratory	1 (100)					1
Totals	36	26	3	3	2	70

Abbreviation: ENT, ear, nose and throat; PEG, percutaneous endoscopic gastrostomy.

**Table 3:** Feeding mode in relation to categorical diagnosis for children receiving an enteral formula with food‐derived ingredients.

Diagnosis category, n (%)	Continuous	Bolus	Combined	Totals
Neurological	20 (54)	15 (40)	2 (5)	37
Cardiac	8 (67)	4 (33)		12
Gastrointestinal	5 (100)			5
Oncology		5 (100)		5
Metabolic	1 (33)	2 (67)		3
Renal disorder	3 (100)			3
Endocrine	1 (50)	1 (50)		2
ENT	2 (100)			2
Respiratory	1 (100)			1
Totals	41	27	2	3

Abbreviation: ENT, ear, nose and throat.

The most common reason for commencing an EFI was the result of a reported gastrointestinal symptom, 58/70 (83%), which was categorised in to six gastrointestinal symptoms (Table 4). The most common gastrointestinal symptom reported before commencing EFI was loose stools in 22 of 58 (38%) children, followed by 15 of 58 (26%) children with constipation. Other reasons for commencing an EFI was as an alternative to a blended diet, when a blended diet was clinically contradicted in eight of 70 (11%); four of 70 (18%) children commenced EFI as the dietitians’ recommended first‐line enteral formula (with no underlying gastrointestinal symptom).

**Table 4:** Outlines the reported gastrointestinal symptom before and after commencing an enteral formula with food‐derived ingredients (within 7 days).

Gastrointestinal Symptom	N (%)	Reported improvement in symptoms within 7 days after feed started, n (%)
**Loose stools**	22 (38)	20/22 (90)
**Constipation**	15 (26)	12/15 (73)
**vomiting**	8 (13)	6/8 (75)
**Abdominal discomfort**	7 (12)	5/7 (71)
**Reflux**	5 (8)	3/5 (60)
**High stoma output**	1 (2)	1 (100)

Within 7 days of commencing an EFI, there was reported improvement in gastrointestinal symptoms in all categories. Over 80% improvement in symptoms reported for vomiting, loose stools and constipation (Table 4).

Fifty‐eight of 70 children were established on an enteral formula and displaying at least one gastrointestinal symptom before commencing an EFI. Of these, 30 of 58 (52%) children were on a whole protein formula, 13 of 58 (23%) children were on a peptide formula and 15 of 58 (25%) children were on an amino acid formula (Table 5). Within 7 days of feed being changed to EFI, clinicians reported that 48 of 58 (82%) children's gastrointestinal symptoms improved. However, 10 of 58 (17%) children did not see any improvement after commencing an EFI and either continued EFI, reverted to pervious enteral formula or changed to a hydrolysed peptide or amino acid formula, with one child requiring parenteral nutrition (Table 5) The feed regimen, feeding route and mode of enteral feeding remained the same before and after an EFI was commenced.

**Table 5:** Outlines enteral formula children were on before changing to enteral formula with food‐derived ingredients and reported improvement in gastrointestinal symptom.

Initial enteral formula before EFI	*n* = 58	Reported improvement in symptoms within 7 days after EFI started	Other outcomes after the EFI formula started
**Whole protein, n (%)**	30 (52)	25/30 (85)	Two required hydrolysed formula
			One required parental nutrition
**Peptide formula, n (%)**	13 (23)	11/13 (88)	One required an amino acid formula
**Amino acid formula, n (%)**	15 (25)	12/15 (80)	Two reverted to amino acid formula

Abbreviation: EFI, enteral formula with food‐derived ingredients.

None of the eight children admitted on a blended diet reported any gastrointestinal symptoms prior to commencing EFI. All eight children continued on EFI until they were discharge from intensive care and re‐commenced blended diet on the step‐down ward.

The mean ± SD feed volume when EFI commenced was 903 ± 318 ml; which equated to a mean ± SD energy intake of 1083 ± 382 kcal/day, 92% ± 12% total energy intake (SACN, 2011)^20^ and 9 g of fibre/day. Data were collected on the 34 of 70 (48%) children who remained on EFI for at least 1 month when they were an inpatient. Comparative analysis was collected for weight‐for‐age *Z*‐score, percentage of total energy intake and energy intake (kcal/day). The comparative analysis reported no significant difference between baseline and 1‐month measurements. The comparative analysis reported no significant difference in weight‐for‐age *Z*‐score, % total energy intake or energy intake (kcal/day) (Table 6).

**Table 6:** Change in energy intake and weight‐for‐age *Z*‐score from baseline to 1 month in children who commenced an enteral formula with food‐derived ingredients.

	Baseline, *n* = 70	1 month, *n* = 34	*p* value
**Feed volume (ml), mean ± SD**	903 ± 318	985 ± 355	0.6
**Daily energy intake (kcal), mean ± SD**	1083 ± 382	1182 ± 324	0.7
**Total energy intake (%), mean ± SD**	92 ± 12	94 ± 8	0.8
**Daily fibre intake (g), mean ± SD**	9 ± 4	10 ± 4	0.8
**Weight‐for‐age** ***Z*** **‐score**	−1.5	−1.6	0.4

At the time of data collection, 23/70 (33%) children had been established on an EFI for 1–6 months, and 14/70 (20%) children established on an EFI for more than 1 year (Table 7).

**Table 7:** Duration (months) children had been on an enteral formula with food‐derived ingredients at the time of data collection, including inpatients and discharged patients.

Duration of children on EFI at the time of data collection,	n (%)
**Less than 1 month**	16 (21)
**Between 1 and 6 months**	23 (33)
**Between 6 and 12 months**	17 (24)
**More than 1 year**	14 (20)

Abbreviation: EFI, enteral formula with food‐derived ingredients.

In total, 42 children were discharged on an EFI. However, before admission, 23/42 (55%) children were already established on home enteral feeding (none were on an EFI prior to admission). Of these, 17/23 (74%) were displaying gastrointestinal symptoms prior to admission and requested to continue EFI on discharge. Therefore, 19/42 (45%) children were new home enteral tube feeders on discharge on EFI.

## DISCUSSION

Commercially available EFIs are relatively novel in the UK, meaning that there is limited evidence to reflect its use and tolerance. Our review suggests that dietitians within a specialist hospital are implementing an EFI in children who are already established on a standard enteral formula displaying gastrointestinal symptoms. We report improved gastrointestinal symptoms in children who had commenced an EFI. Dietitians are also commencing an EFI as a compromise to an enteral blended diet when a blended diet is requested by parents. Dietitians are also using EFI as a first‐line formula in children without underlying gastrointestinal symptoms.

Any potential to improve unpleasant symptoms, to positively impact a child and family's quality of life or medical care during tube feeding should be examined.^10^ In the present study, the most common reason for dietitians to commence an EFI was to improve gastrointestinal symptoms. Of the 40 children who were already established on an enteral formula before commencing EFI, 85% reported an improvement in gastrointestinal symptoms within 7 days of commencing the EFI. These results support a previous publication that observed the impact of switching children from a standard enteral formula who had gastrointestinal symptoms to an EFI. In a previous study, dietitians collected data from 43 medically unwell children and reported an improvement in retching, flatulence, loose stools and constipation.^12^

In our review, no adverse events were reported but 6/40 (15%) children saw no improvement when the formula was switched to EFI and required a peptide or amino acid formula. Of note, the British Dietetic Association Practice Toolkit for The Use of Blended Diet with Enteral Feeding Tubes (Section 4.7.2; 2021) states that children who have been tube fed from birth will not have been through a weaning process and exposed to common allergens. Parents are advised to start by adding 30 ml of blended food (or 30 ml of food‐derived formula) to their current formula and gradually build the volumes to tolerance and nutritional requirements.

Over 50% of children in the present study had a neurological impairment, accounting for most children on long‐term gastrostomy feeding and post‐pyloric jejunal feeding. Feed tolerance is generally worse in children with a neurological impairment, associated with posture and tone disorders and side effects of medications.^18^ Up to 85% of children with severe neurological impairment have feeding disorders and require enteral tube feeding; gastrointestinal symptoms associated with neurological impairment include dysmotility and pain associated with feeding (feed‐induced dystonia).^21^ Many caregivers desire a blended tube feed instead of commercial formula for their child, stating a blended diet is more physiologically normal and reflects familiar family feeds, and ultimately improves gastrointestinal symptoms.^3^ The findings from a retrospective study by Walker et al.^22^ reported transitioning children with significant special healthcare needs from commercial formula to blended tube feed resulted in improvement in gastrointestinal symptoms, reduced the need for gastro‐related medications, supported growth goals and contributed to improved oral feeding. However, commencing an enteral blended diet in an acute clinical setting is not always possible.^12^

Although the exact mechanisms as to why EFIs and blended tube feeds are better tolerated in some children remains unclear. One theory that has been postulated is associated with the amount and mixture of fibre and the subsequent beneficial impact on the gut microbiome.^9^ Non‐digestible dietary fibre undergoes fermentation by the intestinal microbiota to produce short‐chain fatty acids, which positively impact the local and systemic immune system.^23^ The diversity of the gut microbiome is influenced by the variety of the diet; a diet solely of commercial enteral feeds has been implicated in reducing the diversity of microbial species in the gut microbiome^24^; therefore, enteral formulas containing fibre may support normal digestive health.^25^ Commercial tube feeds devoid of fibre appear to negatively alter children's gut microbiome.^26^, ^27^

The average fibre intake in our review was 10 g/day, which meets the recommended daily requirements. Additionally, the EFI contains a mixture of soluble and insoluble fibre (acacia gum, fructo‐oligosaccharides, inulin). An open‐label single‐subject study monitored intestinal short‐chain fatty acids in children admitted to intensive care with sepsis who commenced an EFI. Faecal butyrate and propionate concentrations were maintained whilst feeding on an EFI. The study has highlighted the importance of further research to assess whether an EFI is superior to a standard enteral formula in preserving the intestinal microbiota, thereby mitigating gastrointestinal complications.^28^ The relationship between gut microbiome composition and health has garnered much attention in recent years. Children on a blended tube feed generally had a healthier gut microbiome than their formula‐fed counterparts.^9^

Patients who, during admission, begin to use enteral nutrition and do not recover adequate oral intake need proper planning before discharge. Children with complex medical needs are increasingly cared for at home rather than in the hospital.^29^ Over half of the children in this study who commenced an EFI in the hospital were discharged to their local area on EFI. Hospital discharge describes a point at which inpatient hospital care is completed and care is transferred to the community. This discharge process involves several actions from hospital‐based healthcare professionals, to ensure a patient's transition is safe, efficient and prevents delays. Our hospital has a defined pathway to ensure a smooth transition, all progress and planning is fully documented with a checklist in the patient's electronic record; patients cannot be discharged from the hospital until they have all the necessary equipment.

However, a recently published review of our home enteral tube discharge practice highlights the disparity in the discharge process for patients with newly placed feeding tubes, which impacted a patient's length of stay in the hospital, uncovering the need for further ward training to ensure adherence to Trust guidance and ensure collaboration of services before a patient is discharged to the community.^30^ Of note, the findings from the National Reporting and Learning System and incident data relating to paediatric nasogastric, gastrostomy or jejunostomy feeding at home identified an increasing number of children who require specialist medical care at home. The study identifies a range of safety concerns relating to enteral feeding, highlighting the importance of detailed handovers between hospital and community services; the training of family carers; the provision and expertise of services in the community; and the availability and reliability of equipment.^31^ A meta‐synthesis further echoes the challenges experienced by caregivers, and revealed the need for improved home enteral feeding training for caregivers and psychological support from healthcare professionals, with the aim of providing personalised advice and regimes as part of holistic care.^32^

Blended tube feeds are becoming ubiquitous within paediatric dietetic practice and although the health benefits are evident, the practical implications of implementing a blended tube feed in an acute clinical setting or within an under‐resourced community team may cause some concern even with the available published guidelines.^8^ Therefore, having a commercially available EFI as a compromise or supplement to a blended feed is a welcome addition to the dietitian's repertoire of commercially available enteral formulas. Based on a previous study showing improved gastrointestinal symptoms in children using an EFI ^12^ and data from the present study, the evolving practice of dietitians implementing an EFI at specialist children's hospital is demonstrated.

### Limitations

Retrospective studies have several limitations as a result of their design, which are dependent on the review of records and documentation. Therefore, the results are ungeneralisable and, rather than stating causation, we can only allude to a potential association that an EFI may improve gastrointestinal symptoms. A strength of the present study is its reasonable sample size from a single centre study, and the data were collected from 15 dietitians from several different specialities within a tertiary paediatric centre.

## CONCLUSIONS

In our specialist hospital, an EFI was primarily implemented by dietitians in children with reported gastrointestinal symptoms who were already established on an enteral formula. However, dietitians are increasingly implementing an EFI as their first‐line whole protein enteral formula in children with no underlying gastrointestinal symptoms. Furthermore, in an acute clinical setting, an EFI was implemented as a compromise to an enteral blended diet when requested by parents. To substantiate our findings, we suggest an interventional study that randomises patients to either a blended diet, EFI or standard polymeric formula as a “gold standard” design to allow investigation of clinical and nutritional outcomes.

## AUTHOR CONTRIBUTIONS

**Graeme O'Connor:** Conceptualisation Ideas; formulation of overarching research goals and aims and design of methodology. Writing – Review and Editing. **Angela Camacho Velandia:** Data Curation Management activities to produce research data. **Zoltan Hartfiel Capriles:** Formal analysis – Application of statistics to synthesise study data. Ethical Approval for data collection was granted by Great Ormond Street Hospital Audit, Quality Improvement and Service Evaluation Committee (registration number GOSH2022/3234).

## CONFLICTS OF INTEREST STATEMENT

Dr G. O'Connor reports grants from Nestle Health Science during the conduct of the study. The dietetic department received £40 payment per patient data (£2800). Funds will be used to support dietitians to attend conferences.

### PEER REVIEW

The peer review history for this article is available at https://www.webofscience.com/api/gateway/wos/peer-review/10.1111/jhn.13374.

## TRANSPARENCY DECLARATION

Dr Graeme O'Connor affirms that this manuscript is an honest, accurate, and transparent account of the study being reported; that no important aspects of the study have been omitted; and that any discrepancies from the study as planned (and, if relevant, registered) have been explained.

## Data Availability

The data that support the findings of this study are available from the corresponding author upon reasonable request.
